# Assessing asthma severity based on claims data: a systematic review

**DOI:** 10.1007/s10198-016-0769-2

**Published:** 2016-03-01

**Authors:** Christian Jacob, Jennifer S. Haas, Benno Bechtel, Peter Kardos, Sebastian Braun

**Affiliations:** 1 Xcenda GmbH, Lange Laube 31, 30159 Hannover, Germany; 2 GlaxoSmithKline, Uxbridge, Greater London UK; 3 Group Practice and Centre for Pneumology, Allergy and Sleep Medicine at Red Cross Maingau Hospital, Frankfurt am Main, Germany

**Keywords:** Asthma, Claims data, Exacerbation, Persistent, Intermittent, Systematic review, HEDIS, Leidy, GINA, Economic evaluation

## Abstract

**Introduction:**

Asthma is one of the most common chronic diseases in Germany. Substantial economic evaluation of asthma cost requires knowledge of asthma severity, which is in general not part of claims data. Algorithms need to be defined to use this data source.

**Aims and objectives:**

The aim of this study was to systematically review the international literature to identify algorithms for the stratification of asthma patients according to disease severity based on available information in claims data.

**Methods:**

A systematic literature review was conducted in September 2015 using the DIMDI SmartSearch, a meta search engine including several databases with a national and international scope, e.g. BIOSIS, MEDLINE, and EMBASE. Claims data based studies that categorize asthma patients according to their disease severity were identified.

**Results:**

The systematic research yielded 54 publications assessing asthma severity based on claims data. Thirty-nine studies used a standardized algorithm such as HEDIS, Leidy, the GINA based approach or CACQ. Sixteen publications applied a variety of different criteria for the severity categorisation such as asthma diagnoses, asthma-related drug prescriptions, emergency department visits, and hospitalisations.

**Conclusion:**

There is no best practice method for the categorisation of asthma severity with claims data. Rather, a combination of algorithms seems to be a pragmatic approach. A transfer to the German context is not entirely possible without considering particular conditions associated with German claims data.

## Introduction

Asthma is one of the most common chronic diseases, diagnosed in about 10 % of children and 4–5 % of the adult population in Germany [1]. The economic burden for the German Statutory Health insurance has increased gradually from 2002 to 2008 up to €1,789,000,000 for the year 2008 [2]. The treatment of asthma varies based on the severity of symptoms and disease manifestation. An insufficiently treated asthma patient can suffer from life-threatening asthma attacks with the need for emergency hospitalisation. It is generally accepted that both asthma burden, i.e. for patients in terms of quality of life etc., and treatment costs increase with asthma severity and insufficient control [3]. Substantial economic evaluation of asthma costs requires knowledge of asthma severity, which is generally assessed by using clinical information from the patient. Asthma is a heterogeneous disease whose symptoms can vary over time, and that can change rapidly from day to day. Given that the disease is well-characterized in some patients, the relationship between the underlying disease processes and their clinical manifestations may not be strong. This issue poses a challenge regarding how patients with asthma should be diagnosed and assessed, and how treatment should be adjusted [4]. The concept of asthma severity itself has evolved substantially over the years. Previous Global Initiative for Asthma (GINA) guidelines have differentiated asthma severity into four categories: intermittent, mild persistent, moderate persistent, and severe persistent, referring to the clinical characteristics before treatment and the magnitude of disease features such as the severity of airway obstruction [5]. A patient’s treatment is decided based on this severity classification. As the clinical perspective of asthma has been refined over the years, now focussing more on asthma control rather than on severity, the assessment of severity from a health economic perspective is still of importance given the possibilities of disease management [3]. In general, severity reflects the underlying disease manifestations and thus helps targeted treatments. Furthermore, maintaining a concept of asthma severity includes the option of referring to patients with whom asthma management is challenging either due to poor adherence or, although being adherent, requiring high-intensity treatment [4]. These patients absorb a high proportion of asthma health resources, which is relevant from a health economic perspective.

Hence, not only is the level of asthma control important in terms of the treatment required to achieve adequate asthma treatment, but also the corresponding asthma severity.

Claims data offer important advantages for economic evaluations by providing observational information for a large number of patients, which reflect decisions made both by health care providers in routine clinical practice and by patients with regard to prescription fills and use of inpatient and outpatient care [6]. German claims data include information on an individual patient level such as: biographic data (e.g. age, gender, etc.), healthcare resource utilisation and direct healthcare costs for outpatient and inpatient procedures, drugs, devices and aids, occupational therapies, sick leave payments (with reason) and early retirement. German healthcare insurances cover the most health care services, resulting in only marginal patient co-payments. Healthcare provider payments on the expense of sickness funds (hospital, physician, or pharmacist) represent almost the complete direct health care costs on an individual basis. Due to federal data protection laws, claims data do not include direct clinical data input, such as measures of lung function, forced expiratory volume (FEV) or peak expiratory flow (PEF). Considering that no direct clinical data is captured in claims data, methods to identify different disease severities and disease worsening are needed in order to be able to use this data source for economic evaluation [7]. A variety of algorithms has been developed over the past two decades to fill this gap. Healthcare Effectiveness Data and Information Set (HEDIS) is a quality measurement program from the National Committee for Quality Assurance developed on a claims data based definition of persistent asthma. This definition relies on asthma-coded medical visits and asthma-related pharmacy claims. According to this definition, a population can be identified for whom asthma controller therapy is indicated [8]. In order to be identified as a persistent asthma patient, one or more of the following criteria must be met for the current year: at least one emergency department (ED) visit with asthma as the principal diagnosis, or at least one acute inpatient claim/encounter with asthma as the principal diagnosis, or at least four outpatient asthma visits with asthma as one of the listed diagnoses and at least two asthma medication dispensing events, or at least four asthma medication dispensing events [6, 9, 10]. Recent publications have modified the HEDIS criteria to a 2-year timeframe for the assessment of the above described criteria [11–17]. Although the HEDIS criteria was first used for claims data studies by Berger et al. [18], a validation of the criteria was lacking until 2010. Schatz et al. [8] used survey data including medication use, asthma symptoms and the presence of exacerbations to validate the HEDIS criteria.

The Leidy method [19] determines mild persistent asthma based on the frequency of claims for β_2_-agonist combined with the frequency of claims for oral corticosteroid prescriptions (OCS). Mild persistent asthma is defined by four to six short-acting β_2_-agonist (SABA) refills and zero oral OCS prescriptions per year, or two to three SABA refills and less than two OCS prescriptions per year. Furthermore, one (or less) SABA refill and one oral OCS prescription per year can also account for mild persistent asthma. Moderate persistent asthma includes more than six SABA refills and less than two OCS prescriptions per year, or four to six SABA refills and one to two OCS prescriptions per year. Patients with severe persistent asthma are required to have more than six SABA refills per year and the number of OCS prescriptions per year is greater than or equal to two. Moreover, zero to six SABA refills and three or more SABA prescriptions per year also constitute severe persistent asthma. Clinical validation of the Leidy criteria is warranted [19].

The current GINA guideline provides recommendations for categorizing levels of asthma control. However, previous GINA documents have subdivided asthma by severity based on the level of symptoms, airflow limitation, and lung function variability. Four categories were included: intermittent, mild persistent, moderate persistent, and severe persistent [20]. The daily dose of inhaled corticosteroids (ICS) and long-acting β_2_-agonist LABA were divided into low and high intensity treatment. Mild persistent asthma was defined by either using low-dose ICS consistently, or using ICS inconsistently, including zero to two claims. Patients with moderate persistent asthma were defined as such if they received low-dose ICSs and either a LABA, a leukotriene modifier, theophylline or medium- or high-dose ICSs. Severe persistent asthma was defined by the use of medium- or high-dose ICS plus a LABA along with other controllers [20]. A validation of the GINA based claims data algorithms is still lacking.

The Canadian Asthma Consensus Guideline (CACQ)-based database indexes were developed and validated by Firoozi et al. [21]. The severity index defines three levels of asthma severity by assessing asthma medication and the presence of moderate/severe asthma exacerbations over a period of 1 year. Patients in the mild asthma category are supposed to show no presence of moderate/severe asthma exacerbations over a period of 1 year, receive ICS doses of 0–500 µg/day with no additional controller therapy or, for patients with additional controller therapy, a dosage of 0–250 µg ICS per day. Moderate asthma is classified by ICS doses of >500 µg/day for patients without additional controller therapy, and doses of >250 µg/day for those with additional controller therapy. Patients with high use of SABA and moderate or severe asthma exacerbations are also classified as moderate asthma. The category of severe asthma consists of individuals receiving ICS doses of >1000 µg/day, or >10 doses of SABA per week, with moderate/severe exacerbations. The CACQ database indexes were validated against pulmonary function test results of a sample of 71 randomly selected asthma patients. Patients were recruited from two asthma clinics and medical chart reviews were used to validate the CACQ database indexes against FEV_1_ values [21].

The aim of this study was to systematically review the international literature to assess if the already existing algorithms are applied for the stratification of asthma patients according to disease severity based on available information in claims data. Furthermore, potential best practice standards are identified and their transferability to the German setting was discussed.

## Methods

### Data sources

A systematic literature review was performed in July 2015 using DIMDI SmartSearch—a search engine including several databases with a national and international scope, e.g. BIOSIS, MEDLINE, and EMBASE. The database search was performed on 1 July 2015 and included all publications present at that date in the included databases. An update of the search was performed on 24 September 2015. No further timely restrictions were applied. Additionally, a manual search was conducted to track references quoted by relevant articles. The review was limited to publications in the English and German languages. The systematic search was broadly defined to be able to identify a variety of publications. A three-step approach was used to identify publications that classified the severity of asthma by utilizing claims data. Asthma-specific publications were searched by focussing on publications mentioning the search term “asthma” in the abstract. English and German synonyms for claims data (“Abrechnungsdaten” eng. “administrative data” or “claims data”, “Routinedaten” eng. “routine data”, and “Sekundärdaten” eng. “secondary data”) in full text search were used to identify relevant claims-data-based publications. Asthma-specific severity search terms were used in full text search (“schwer” eng.“sever”, “mild”, “persistierend” eng. “persistent”, “intermittierend” eng. “intermittent”, and “moderat” eng. “moderate”) to focus the search on disease severity. The detailed search algorithm is presented in Table 1.

**Table 1:** Systematic database search

No.	Search term	Results
[1]	ME05, BA05, EA08, EM05, GA03, GM03, IS05	45,263,126
[2]	AB = ?asthma?	199,791
[3]	FT = ?Abrechnungsdaten?	181
[4]	FT = ?Routinedaten?	522
[5]	FT = ?Sekundärdaten?	168
[6]	FT = ?routine data?	2767
[7]	FT = ?administrative data?	23,641
[8]	FT = ?secondary data?	10,396
[9]	FT = ?claims data?	23,272
[10]	[3] OR [4] OR [5] OR [6] OR [7] OR [8] OR [9]	58,780
[11]	FT = ?sever?	5,032,732
[12]	FT = ?mild?	684,188
[13]	FT = ?persistent?	417,821
[14]	FT = ?intermittent?	146,361
[15]	FT = ?moderate?	953,986
[16]	FT = ?schwer?	143,716
[17]	FT = ?persistierend?	3159
[18]	FT = ?intermittierend?	1891
[19]	FT = ?moderat?	981,340
[20]	[11] OR [12] OR [13] OR [14] OR [15] OR [16] OR [17] OR [18] OR [19]	6,476,060
[21]	[2] AND [10] AND [20]	640

*ME05* MEDLINE, *BA05* BIOSIS Previews, *EA08* EMBASE Alert, *EM05* EMBASE, *GA03* gms, *GM03* gms Meetings, *IS05* SciSearch, *AB* search Abstract, *FT* search Freitext (engl. full text), *?* wildcard

The systematic search yielded a total of 640 publications, of which 335 were excluded as duplicates. Titles and abstracts of potential studies were separately screened by three independent reviewers. We excluded studies upfront that did not focus on asthma or did not use claims data. Publications were excluded if no full text was available, e.g. poster presentations and conference abstracts. Based on a full text review, studies were excluded if they did not apply an algorithm to distinguish between different disease severities or did not describe the methodology of identifying asthma disease severities.

## Results

### Asthma disease severity

**Fig. 1:**
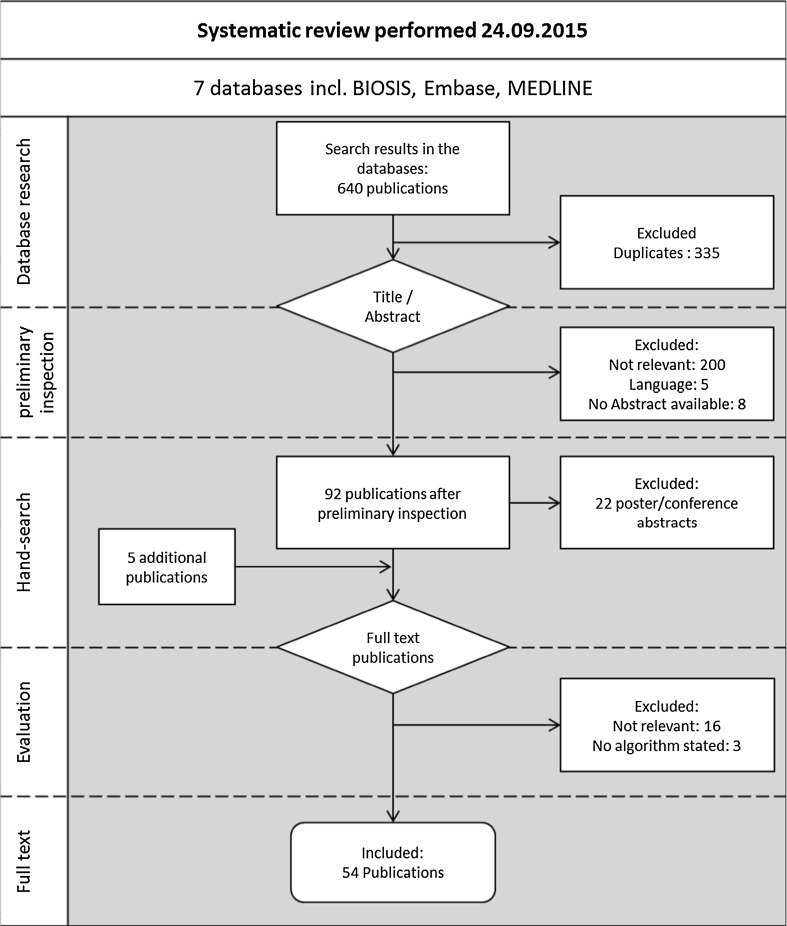
Study selection

The systematic research yielded 54 publications assessing asthma severity based on claims data (Fig. 1). Out of the 54 identified studies, 45 were conducted in the United States, 5 in Canada, 1 in Finland, 1 in Germany, 1 in New Zealand, and 1 in Puerto Rico. The identified studies were categorised based on the assessed asthma severity. Thirty-nine publications referred to either HEDIS criteria, Leidy criteria, the GINA guideline-based approach or the CACQ-based database indexes. An overview of the criteria of four specific algorithms that were applied throughout the publications is presented in Table 2.

**Table 2:** Algorithms to identify asthma severity

Criteria/method	Description
HEDIS criteria	Persistent asthma: 12-month-period
	≥One acute inpatient hospitalisation with asthma as a primary diagnosis OR
	≥One ED visit with a primary asthma diagnosis OR
	≥Four claims for asthma prescription medications dispensed OR
	≥Four outpatient visits with asthma listed anywhere as one of the diagnosis AND
	≥Two claims for asthma prescription medications including quick-relief medications, controllers, biologic agents, and systemic corticosteroids
Leidy criteria	Mild intermittent asthma
	≤One inhaled β_2_-agonist prescription and no oral steroid prescription per year
	Mild persistent asthma
	≤One inhaled β_2_-agonist prescription and one oral steroid prescription per year OR
	Two or three inhaled β_2_-agonist use per year and two oral steroid prescriptions per year OR
	Four to six inhaled β_2_-agonist canister use per year and zero oral steroid prescriptions per year OR
	Moderate persistent asthma
	≤One inhaled β_2_-agonist prescription and two oral steroid prescription per year
	Four to six inhaled β_2_-agonist canister use per year and two oral steroid prescriptions per year OR
	>Six prescriptions of inhaled β_2_-agonist per year and less than two oral steroid prescriptions per year
	Severe persistent asthma
	≤One inhaled β_2_-agonist prescription and more than two oral steroid prescriptions per year
	Two or three inhaled β_2_-agonist use per year and more than two oral steroid prescriptions per year OR
	Four to six inhaled β_2_-agonist canister use per year and more than two oral steroid prescriptions per year OR
	>Six prescriptions of inhaled β_2_-agonist per year and more than one oral steroid prescriptions per year
GINA criteria	Mild persistent asthma
	Low dose ICS
	Moderate persistent asthma
	Medium dose of ICS OR
	Low-medium dose ICS with LABA
	Severe persistent asthma
	High-dose ICS with or without LABA
CACQ database indexes	Mild asthma
	0–500 µg ICS per day, no other controller therapy, 0–3 doses of SABA per week, no moderate to severe exacerbations^a^
	0–250 µg ICS per day, further controller therapy, 0–3 doses of SABA per week, no moderate to severe exacerbations^a^
	0–500 µg ICS per day, no other controller therapy, 0–3 doses of SABA per week, moderate to severe exacerbations^b^
	0–250 µg ICS per day, further controller therapy, 4–10 doses of SABA per week, no moderate to severe exacerbations^b^
	0–500 µg ICS per day, no other controller therapy, 4–10 doses of SABA per week, no moderate to severe exacerbations^b^
	Moderate asthma
	251–500 µg ICS per day, further controller therapy, 0–10 doses of SABA per week, no moderate to severe exacerbations^a^
	501-1000 µg ICS per day, 0–10 doses of SABA per week, no moderate to severe exacerbations^a^
	>1000 µg ICS per day, 0–3 doses of SABA per week, no moderate to severe exacerbations^a^
	0–250 µg ICS per day, further controller therapy, 4–10 doses of SABA per week, moderate to severe exacerbations^b^
	0–500 µg ICS per day, no other controller therapy, 4–10 doses of SABA per week, moderate to severe exacerbations^b^
	0–250 µg ICS per day, further controller therapy, >10 doses of SABA per week, no moderate to severe exacerbations^b^
	0–500 µg ICS per day, no other controller therapy, >10 doses of SABA per week, no moderate to severe exacerbations^b^
	251–500 µg ICS per day, further controller therapy, >10 doses of SABA per week, no moderate to severe exacerbations^b^
	251–500 µg ICS per day, further controller therapy, 0–10 doses of SABA per week, moderate to severe exacerbations^b^
	501–1000 µg ICS per day, >10 doses of SABA per week, no moderate to severe exacerbations^b^
	501–1000 µg ICS per day, 0–10 doses of SABA per week, moderate to severe exacerbations^b^
	Severe asthma
	Controlled
	>1000 µg ICS per day, 4–10 doses of SABA per week, no moderate to severe exacerbations^a^
	0–1000 µg ICS per day, >10 doses of SABA per week, moderate to severe exacerbations^b^
	>1000 µg ICS per day, 0–10 doses of SABA per week, moderate to severe exacerbations^b^
	>1000 µg ICS per day, >10 doses of SABA per week^b^

* HEDIS* Healthcare Effectiveness Data and Information Set, *CACQ* Canadian Asthma Consensus Guidelines, *GINA* Global Initiative for Asthma, *ICS* inhaled corticosteroid, *LABA* long-acting beta-agonist, *SABA* short-acting β_2_ agonist

^a^Controlled

^b^Uncontrolled

As Table 3 shows, of the 39 publications, most of the identified studies (31 publications) used the HEDIS criteria to identify patients with persistent asthma. Of these, seven publications used the 2-year version, while 6 out of the 39 publications used a combination of three specific algorithms, namely HEDIS 1-year, Leidy and GINA. As GINA only refers to asthma control it was not applied as single criteria whereas Leidy was applied in three studies. The CACQ database indexes were applied in five publications.

**Table 3:** Overview of the studies referring to algorithms (Methods/ criteria) to identify asthma severity

Reference	HEDIS 1-year	HEDIS 2-year	Leidy	GINA	CACG
Andrews et al. [22]	X				
Baxter et al. [23]	X				
Berger et al. [18]	X				
Broder et al. [9]	X				
Cabana et al. [24]	X				
Canino et al. [25]	X				
Dombkowski et al. [26]	X				
Finkelstein et al. [27]	X				
Fuhlbrigge et al. [28]	X				
Hsu et al. [29]	X				
Mosen et al. [30]	X				
Richardson et al. [31]	X				
Schatz et al. [32]	X				
Schatz et al. [33]	X				
Schatz et al. [34]	X				
Schatz et al. [8]	X				
Wakefield & Cloutier [35]	X				
Wilson et al. [36]	X				
Birnbaum et al. [6]	X		X	X	
Colice et al. [37]	X		X	X	
Colice et al. [38]	X		X	X	
Colice et al. [10]	X		X	X	
Ivanova et al. [39]	X		X	X	
Ivanova et al. [40]	X		X	X	
Dombkowski et al. [14]		X			
Schatz and Zeiger [11]		X			
Schatz et al. [17]		X			
Vernaccio et al. [15]		X			
Yong and Werner [12]		X			
Yoon et al. [13]		X			
Zeiger et al. [16]		X			
Allen-Ramey et al. [42]			X		
Erickson et al. [43]			X		
Wells et al. [44]			X		
Blais and Beauchesne [45]					X
Blais et al. [46]					X
Blais et al. [47]					X
Firoozi et al. [21]					X
Firoozi et al. [48]					X

The use of HEDIS, Leidy and GINA was applied as a three-fold process. The six identified publications stated that HEDIS measures might mislabel mild intermittent asthma as mild persistent asthma, so additionally patients were required to meet the Leidy criteria [19]. Furthermore, Leidy and a GINA-guideline-based approach were then incorporated in conjunction into the algorithm for the confirmation of mild persistent asthma [6].

Sixteen publications applied a variety of different criteria for the severity categorisation and did not use the specific algorithms described above. Of these, 11 publications identified patients with a milder form of asthma that differentiated between classifying patients as “mild”, “mild intermittent”, and “intermittent”. Seven publications identified patients with more severe forms of asthma. Distinctions were drawn between “persistent”, “mild persistent”, “moderate persistent”, “severe persistent” and “severe” asthma. The different severity categories were assessed according to their own design. Out of the 16 publications, two studies investigated “low-risk” and “high-risk” asthma.

Table 4 presents an overview of the distribution of severities evaluated according to their own distinct definitions.

**Table 4:** Evaluated severities of asthma

Reference	Mild	Mild Inter-mittent	Inter-mittent	Moderate	Persistent	Mild Persistent	Moderate Persistent	Severe Persistent	Severe	Low-risk	High-risk
Friedman et al. [49]	X										
Friedman et al. [50]	X										
Friedman and Yawn [51]	X										
Navaratnam et al. [52]	X										
Navaratnam et al. [53]	X										
Navaratnam et al. [54]	X										
Erickson et al. [43]	X			X					X		
Friedman et al. [55]	X								X		
Gillies et al. [56]		X									
Guo et al. [57]		X				X	X	X			
Jacob et al. [58]			X		X						
Rust et al. [59]					X						
Vaidya et al. [60]					X						
Wertz et al. [61]							X	X			
Klemets et al. [62]										X	X
Talbot et al. [63]										X	X

Based on the variety of assessed severities and the methods applied for their categorisation, all publications were stratified according to the determined severity.

### Mild asthma

The International Classification of Diseases, Ninth Revision, Clinical Modification (ICD-9-CM) codes 493.0X, 493.1X, or 493.9X were applied throughout all publications as an appropriate tool for the inclusion of patients with asthma. Differences were linked to the number of claims being coded for each patient during the analysis. Friedman et al. [51] classified patients as having mild asthma based on no recorded exacerbations, which was defined as an asthma episode that required hospitalisation, an ED visit, or an outpatient visit in which patients received nebulised medication or an OCS prescription, and use of less than two canisters of inhaled SABA in the 6 month pre-index period. Patients were required to have at least one prescription for any dose of fluticasone/salmeterol (FPS) fixed dose combination, which was then considered the index date.

Another study from Friedman et al. [55] assessed mild asthma based on one index prescription of fluticasone propionate/salmeterol and no further ICS use. The authors determined severe persistent asthma extensively (see severe persistent asthma section of this paper) and considered patients as having mild asthma if none of the severe criteria was met.

In a study from Friedman et al. published in 2010 [49], all patients were required to be enrolled in a health plan for at least 1 year before and after the index date, which was defined as the first prescription fill of either mometasone furoate delivered through a dry powder inhaler or fluticasone propionate. Moreover, patients received no ICS/SABA combination therapy within 7 days of the index date. Patients were defined as having mild asthma if they had no asthma-related exacerbation, defined as described above, and less than three SABA canister claims during the pre-index period.

In a study by Friedman et al. [50], patients were required to be enrolled on their health plan for at least 1 year before and after their index date, and with no prior claims for asthma exacerbation. The index date was defined as a first claim for either mometasone furoate or beclomethasone dipropionate prescription. The classification of mild asthma was made based on less than three SABA canister claims and no asthma exacerbation, defined as an asthma episode that required hospitalisation, an ED visit, or an outpatient visit with nebulised medication or a prescription for OCS, within 12 months prior to index date.

Navaratnam et al. [54] defined mild asthma as less than or equal to two SABA canister claims and no exacerbation, which was defined as an asthma episode that required hospitalisation, an emergency department visit, or an outpatient visit with nebulised medication or an OCS prescription, during the pre-index period. Patients were required to be enrolled at least 1 year prior and after the index date, which was defined as the first prescription for mometasone furoate or fluticasone propionate with salmeterol. The same criteria were applied in further studies published by Navaratnam et al. [52, 53].

Erickson et al. [43] defined mild asthma based on the Leidy criteria for “mild intermittent” asthma and “mild” asthma using a method described by Cai et al. [64]. Mild intermittent asthma is defined as one or less canisters of an inhaled β-agonist within 12 months, and mild asthma as five or less prescriptions of inhaled or oral β-agonist, a theophylline compound, inhaled ipratropium bromide, or an anti-allergic compound (cromolyn, nedocromil or ketotifen).

### Mild intermittent asthma

Gillies et al. [56] defined four asthma treatment groups with a diagnosis of asthma. Three steps were described, adopted from the British Guideline of the Management of Asthma. Step 1, mild intermittent asthma, was defined by at least two SABA inhalers dispensed in a 12-month period. Step 2 and 3 comprised a more severe form of asthma and the corresponding treatment.

Guo et al. [57] divided asthma severity into four levels: mild intermittent, mild persistent, moderate persistent, and severe persistent asthma. Patients were identified based on an asthma diagnosis indicated by an ICD-9 Code 493.xx from an institutional or medical claim. The severity assessment was based on the recommended drug regimens from 2002 National Asthma Education and Prevention Program (NAEPP) guidelines update to Expert Panel Report 2 (EPR-2). All patients were required to have a SABA prescription. Whereas patients identified as mild intermittent did not have any claims for an ICS.

### Intermittent asthma

Jacob et al. [58] stratified patients into two mutually exclusive groups of patients with intermittent or persistent asthma. The stratification was based on prescribed asthma medication. Asthma patients were identified by ICD-10-GM codes. All asthma patients without any evidence of asthma medication and those with a record of reliever medication (i.e. at least one prescription of a short acting b2-agonist) were classified as intermittent asthma, if they had no record of an asthma-related hospitalisation in the study period.

### Moderate asthma

Erickson et al. [43] defined moderate asthma based on drugs dispensed within 12 consecutive months. Referring to the frequency of pharmacy claims for multiple combinations of reliever and controller medications, subjects were required to have four to six prescriptions (or canisters) of inhaled β-2 agonist, and/or two prescriptions of oral steroids. Furthermore, patients were classified as moderate if they did not meet the criteria for mild or severe asthma.

### Persistent asthma

Jacob et al. [58] identified patients with persistent asthma by using medication claims for long-acting β-2 agonists (LABA), leukotriene modifiers (LTRA), inhaled corticosteroids, oral corticosteroids, Anti-IgE, theophylline, and ipratropium bromide in combination with hospitalisations with a primary diagnosis of asthma. Patients were classified as having persistent asthma if they had one or more of the mentioned medication claims, or an asthma-related hospitalisation.

Rust et al. [59] assessed patients with at least one inpatient or two outpatient claims for asthma in 2007. Only children aged 5–12 years with an initial claim for an inhaled corticosteroid prescription were included. The initial claim was defined by no record of long-term control prescription drug claims, including inhaled corticosteroids, leukotriene inhibitors and oral corticosteroids, in the 90 days prior to the initial claim with ICS. Children with their initial ICS claim during the period from 1 April 2007 to 30 September 2007 were staged as “persistent” or described as having asthma of sufficient severity and persistence to require ICS as a long-term controller medication. More severe asthma was defined by drug claims for two or more SABA rescue inhalers within the 90-day period prior to initial ICS prescription. The use of SABA was found to be one of the strongest predictors of asthma-related ED visits among patients who met HEDIS criteria for persistent asthma.

Vaidya et al. [60] referred to patients receiving controller therapy by assessing the prescription drug records, which were required to include at least one claim for inhaled corticosteroids, cromolyn, or montelukast, and were coded between June 2006 and June 2007. Based on an ICD-9-CM code 493.xx in the primary or secondary diagnosis field, subjects with persistent asthma were identified from the outpatient claims from January 2006 to December 2007. The first claim for controller medication was considered the index date, and the 6 months before and after were assessed in the study.

### Mild persistent asthma

Guo et al. [57] identified mild persistent asthma in two groups of patients based on pharmaceutical use. Patients below the age of 5 years were classified as mild persistent by medication regimens over a period of at least 90 days after the asthma index date. Mild persistent asthma included use of SABA, low dose ICS, mast cell stabilizer or leukotriene modifier as an alternative treatment to ICS. Patients above the age of 5 years were classified as mild persistent similar to the drug regimens of children younger than 5 years with the addition of Theophylline as an alternative to ICS treatment.

### Moderate/severe persistent asthma

Guo et al. [57] identified moderate persistent asthma patients by the use of asthma medication within a timeframe of at least 90 days. Patients receiving SABA, low dose of ICS, LABA, leukotriene modifier, and/or Theophylline therapy were classified as moderate persistent asthma patients.

Wertz et al. [61] identified moderate to severe asthma based on at least one medical claim for asthma ICD-9-CM code 493.xx, and at least one pharmacy claim for an asthma controller medication, defined by an Expert Panel [38], such as inhaled corticosteroid monotherapy, leukotriene antagonist, or inhaled corticosteroid combination, between 1 September 2005, and 31 August 2006.

### Severe persistent asthma

Guo et al. [57] identified severe persistent asthma patients by the use of asthma medication within a timeframe of at least 90 days. Patients receiving oral corticosteroids besides their SABA, ICS and/or LABA therapy were classified as severe persistent asthma patients.

### Severe asthma

Erickson et al. [43] observed a variation in the distribution of health-related quality of life and work performance scale scores based on different methods of determining asthma severity. An asthma service claim with a prescribed asthma medication or a claim for two asthma medication prescriptions in the 18 months prior to the survey were considered the basic inclusion criteria for patients with moderate to severe asthma, followed by multi drug use. Multi drug use was defined to categorize severe asthma. If subjects received any of the following groupings in a period of 12 consecutive months before the survey, they were considered to have severe asthma:

- Group 1: at least six canisters or prescriptions of any bronchodilators (inhaled, oral or nebulised β-agonist, theophylline, ipratropium) and at least six more prescriptions for an inhaled corticosteroid or anti-allergic compound
- Group 2: at least three prescriptions in each of at least three different classes of asthma medication, the classes being β-agonist, theophylline, anti-allergic, ipratropium bromide, corticosteroids whether inhaled or oral
- Group 3: at least 2 prescriptions for oral corticosteroids and six or more prescriptions for any other asthma medication
- Group 4: at least 25 canisters of a β-agonist bronchodilator [43].

Friedman et al. [55] classified asthma as severe disease if patients met one of the following criteria within 1 year: one or more claims for an ICS, >365 doses of albuterol from an inhaler or >365 inhalation unit doses of albuterol or levalbuterol, one or more claims for oral corticosteroids (OCSs), an asthma related visit in urgent-care (UC) or in an ED followed by a prescription for an OCS within 7 days of the visit, or a hospital admission for asthma.

### High-risk asthma

High risk asthma was defined by Klemets et al. [62] as a record of at least one asthma related hospitalisation with an ICD code in primary position in a period of 12 months. Talbot et al. [63] modified the definition by adding asthma-related visits to emergency departments and prescriptions of corticosteroids as rescue therapy or long-term courses of oral corticosteroids or prescriptions for three or more β-agonists during the course of 1 year to classify individuals as high-risk asthma patients. Both Klemets et al. and Talbot et al. defined low-risk asthma as not meeting the criteria for high-risk asthma [62, 63].

Table 5 gives an overview of the different severity criteria used in the identified publications. Each of the 16 identified publications not using one of the established algorithms were grouped according to the applied algorithm in terms of the criteria they used to classify asthma severity. The established algorithms were represented by Schatz et al. for HEDIS, Erickson et al. applying Leidy, Birnbaum et al. for GINA, and Firoozi et al. for CACQ [6, 8, 21, 43].

**Table 5:** Severity criteria of asthma

Criteria	Mild	Mild Inter-mittent	Inter-mittent	Moderate	Persistent	Mild Persistent	Moderate Persistent	Severe Persistent	Severe	Low-risk	High-risk
SABA	[49] [21,50-55]	[56,57]	[58]	[21]	[58, 59]	[57]	[57]	[57]	[21]	[63]	
LABA					[58]	[6, 57]	[6, 57]	[6, 57]			
inhaled β-2 agonist	[43]	[43]			[58]	[43]	[43]	[43]	[43]	[63]	[63]
ICS	[21]			[21] [61]	[58] [59, 60]	[6, 57]	[6, 57]	[6] [57]	[21, 43, 61]		[63]
OCS	[21]			[21]	[58]	[43]	[43]	[43, 61]	[21, 43]		[63]
Anti-IgE					[58]						
Theophylline	[43, 65]				[58]		[57]		[43, 65]		
Leukotriene modifier				[61]	[58]	[57]	[57]		[61]		
Ipratropium bromide	[43, 65]				[58]				[43]		
Mast cell stabilizer					[60]	[57]					
Other controller medication	[21]			[21]							
Any Asthma medication					[8]				[43]		
Anti-allergic compound	[43]								[43]		
Outpatient visits					[8]						
No outpatient visits with nebulized medication or OCS prescription	[49-55]										
ED	[21]			[8]					[21]		[62, 63]
No ED	[49-55]									[62, 63]	
Hospitalization	[21]			[21]	[58]				[21]		[62, 63]
No hospitalization	[49-55]		[58]							[62, 63]	
No ICS, OCS,										[63]	

## Discussion

The systematic literature search yielded 54 publications that evaluated asthma severity based on claims data, despite the fact that clinical data is missing in this data source [40]. Different approaches have been developed over the last two decades to overcome this limitation. Previous work has shown that claims-data-based instruments are feasible to assess quality-of-care [33], and that algorithm-based severity categorisation is possible [6]. Claims data analyses provide relevant observational information for a large number of patients, reflecting real-life treatment patterns [40, 66]. The reviewed literature suggests that previously described algorithms such as HEDIS, Leidy and CACG are used widely but no best practice for the identification of disease severity in asthma patients using claims data has been established so far. Also, the HEDIS criteria was applied in 31 publications, but a more differentiated look at the most recent publications indicates that alternatives are still of interest. In the timeframe of the most recent 5 years (2011–2015), six publications used the HEDIS criteria whereas five publications used other algorithms. Expanding the timeframe to the most recent 6 years shifts the result in favor of other algorithms than HEDIS (11 other vs. 10 HEDIS). HEDIS relies on asthma claims coded at ED visits, hospitalisations, outpatient visits, or SABA prescription fills, which is a commonality also found in Leidy’s algorithm. As Birnbaum et al. [6] state, this medication-derived method can categorise patients as having more severe asthma than the symptom-derived methods based on clinical data. An analysis in children with asthma suggested that HEDIS criteria for persistent asthma is very sensitive, but has relatively low specificity; hence, it might misclassify patients with intermittent asthma as having persistent asthma [67]. To avoid this possible misclassification, Leidy’s criteria was applied to exclude patients who might have intermittent asthma, by incorporating minimal requirements for the number of SABA claims to be identified as persistent. Thus, Leidy’s algorithm is commonly used as an additional secondary screen to the HEDIS criteria, when classifying patients with mild persistent asthma [10]. The claims-data-based GINA criteria—an approach used in combination with HEDIS and Leidy—provides recommendations based on the daily dose of ICS and LABA, but is less specific than HEDIS and Leidy when comparing the requirements for asthma medication use based on claims data. However, the GINA guideline is considered the gold standard in clinical practice for the assessment of disease control. In contrast, Leidy’s criteria refers only to SABA use, which does not include inhaled corticosteroids, present in the former GINA guideline [20]. The CACQ database indexes were used as standalone classification for asthma severity, also incorporating asthma control. These indexes use a comprehensive matrix of criteria, including daily ICS dose, weekly SABA dose, other controller medication and markers of moderate/severe exacerbations to assess asthma severity [21]. Thus, they are more complex then HEDIS, LEIDY and GINA. So far a validation for HEDIS, Leidy and CACQ is warranted.

One objective of this review was the evaluation of a potential replication of the identified algorithms to the German setting. Most of the studies presented here were conducted in the United States, where a different health care system and coding system is in place, which makes the assessment of a possible transfer to a German setting even more important. Claims data from the German Statutory Health Insurance are collected primarily for the purpose of reimbursement and documentation. Clinical data, and also information about the intention of the physician, e.g. prescribed dosage and frequency, is missing. This limits the potential of dosage-based classifications such as the GINA-based approach.

In Germany, information on diagnosis in the outpatient sector is given only on a quarterly basis. In contrast, medical services are recorded on a daily basis. Therefore, it is not possible to exactly match a diagnosis with a specific outpatient visit. The limitation also takes effect on the identification of outpatient emergency cases [7]. To apply the HEDIS criteria to German claims data is not completely possible due to the quarterly documentation of outpatient diagnoses. Each diagnosis is only recorded once a quarter for every physician the patient consulted. Especially, the identification of at least four outpatient visits with asthma listed as a diagnosis poses a challenge, since a patient would be required to have an outpatient claim in each quarter or with different physicians to amount to four claims for asthma. Furthermore, the analysis of an emergency case with an asthma diagnosis is possible only for the inpatient setting. Emergency cases in the outpatient setting might be misclassified in terms of multi-morbid patients, due to the quarterly documentation of outpatient diagnoses.

Leidy’s criteria assess asthma based on requirements for the amount of asthma-specific prescriptions per year. Mild persistent asthma is defined by four to six SABA refills and zero oral OCS prescriptions per year or two to three SABA refills and less than two OCS prescriptions per year. This specific algorithm can be applied to German claims data, as the medication prescriptions are documented and can be assessed.

The former GINA guideline provides recommendations for categorising mild or severe persistent asthma based on the daily dose of inhaled corticosteroids and at least a second controller, i.e. LABA, LTRA, Theophylline, and OCS. These criteria cannot be transferred to German claims data without inaccuracy, as the daily dose can only be estimated. The data does not include prescribed dosage information, which might modify the use of ICS and salmeterol in a specific case [7].

The studies that did not refer to the algorithms mentioned above were categorized based on the severity assessed. In total, 16 publications were stratified to mild, mild intermittent, intermittent, moderate, persistent, mild persistent, moderate persistent, severe persistent, severe and low/high-risk asthma. The basis for the inclusion of persistent asthma patients were mostly asthma service claims. The publications varied especially in specificity concerning the amount of prescriptions for asthma specific medication and where asthma claims needed to be coded, i.e. inpatient or outpatient sector, or ED visit [43, 55, 61]. Publications assessing mild asthma with various criteria excluded asthma exacerbations, mostly defined as an asthma episode that required hospitalisation, an emergency department visit, or an outpatient visit in which patients received nebulised medication or a prescription for OCS. Moreover, similar to the partly medication-derived algorithms from HEDIS and Leidy, asthma-specific medication, or an overreliance on SABA, were considered an indication for a higher asthma severity [49–51, 55]. Publications determining moderate to severe persistent asthma focussed on asthma-specific medication, such as fluticasone propionate/salmeterol, albuterol, and levalbuterol, which are β_2_-agonists. Furthermore, the number of prescription fills for inhaled corticosteroids was considered an important identification criterion for more severe asthma [43, 55].

The evaluation of methods applied suggests that asthma severity in administrative data is connected with claims for asthma and asthma-specific medication, varying by the type of therapy received. Claims for oral or inhalative corticosteroids are associated with higher disease severity, whereas mild asthma is associated mostly with restricted use of short-acting β_2_-agonists.

Due to the fact that the identified algorithms have commonalities with the specific algorithms from HEDIS and Leidy, transfer to the German context is possible with a few restrictions. As already mentioned, physician contacts and emergency cases in the outpatient setting cannot be accurately connected to a specific ICD-10-GM diagnosis code. Furthermore, the severity categorisation based on medication use can be applied if the use does not refer to daily doses but instead to the number of prescriptions. It should be noted that claims for prescriptions dispensed can be imprecise as the data identifies only that a canister was dispensed by a pharmacy—regardless of whether the medication was actually used by the patient [27].

## Conclusion

The results of this systematic review suggest that there is no best practice method for the categorisation of asthma severity grades with claims data. Also, although HEDIS is used in the majority of studies, this is more heterogeneous for the most recent publications (2010–2015). Rather, a combination of the specific algorithms seems to be a pragmatic approach. Furthermore, it should be noted that, by the date of the systematic search, only one study was identified that used a study design similar to the German context. The analysis of the specific algorithms indicates some limitations, which might lead to a misclassification of asthma severity if only a single algorithm is applied. A factor common to the assessed algorithms, both specific and unspecific, is that they refer to either asthma-specific medication and/or claims in the inpatient or outpatient sector. It should be noted that the studies vary in the amount of necessary prescriptions for asthma specific-medication and claims in the inpatient or outpatient sector. The transfer to a German context is not entirely possible without considering particular conditions associated with German claims data, especially in the outpatient sector. Nevertheless, as claims data has important advantages based on the observational information for a large number of patients, which also accurately reflects the resource use and costs of a disease, these algorithms could be modified and applied to the German setting and provide an approach for a health economic evaluation.
